# Lectin Digestibility and Stability of Elderberry Antioxidants to Heat Treatment In Vitro

**DOI:** 10.3390/molecules22010095

**Published:** 2017-01-06

**Authors:** Pilar Jiménez, Patricia Cabrero, Damian Cordoba-Diaz, Manuel Cordoba-Diaz, Manuel Garrosa, Tomás Girbés

**Affiliations:** 1 Nutrición y Bromatología, Facultad de Medicina, Universidad de Valladolid, Valladolid 47005, Spain; pilarj@bio.uva.es (P.J.); patricia_cabrero@hotmail.com (P.C.); 2 Farmacia y Tecnología Farmacéutica, Facultad de Farmacia and Instituto Universitario de Farmacia Industrial (IUFI), Universidad Complutense de Madrid, Madrid 28040, Spain; damianco@farm.ucm.es (D.C.-D.); mcordoba@farm.ucm.es (M.C.-D.); 3 Biología Celular, Histología y Farmacología, Facultad de Medicina and Instituto de Neurociencias de Castilla y León (INCYL), Universidad de Valladolid, Valladolid 47005, Spain; garrosa@med.uva.es

**Keywords:** *Sambucus nigra*, elderberry, lectin, ribosome-inactivating protein, polyphenol, free-radicals

## Abstract

Elderberry contains healthy low molecular weight nutraceuticals and lectins which are sequence-related to the elderberry allergen Sam n1. Some of these lectins are type II ribosome-inactivating proteins. The sensitivity of native lectins present in elderberry fruits and bark to the proteolysis triggered by in vitro simulated gastric and duodenal fluids has been investigated. It was found that these lectins are refractory to proteolysis. Nonetheless, incubation for 5–10 min in a boiling water bath completely sensitized them to the hydrolytic enzymes in vitro. Under these conditions neither total Folin-Ciocalteau’s reagent reactive compounds, total anthocyanins and the mixture of cyanidin-3-glucoside plus cyanidin-3-sambubioside, nor antioxidant and free-radical scavenging activities were affected by more than 10% for incubations of up to 20 min. Therefore, short-time heat treatment reduces potential allergy-related risks deriving from elderberry consumption without seriously affecting its properties as an antioxidant and free-radical scavenging food.

## 1. Introduction

Berry consumption is assumed to be healthy presumably because of the high content of antioxidant compounds, mainly phenolic species, that might be considered functional nutrients [1,2]. Anthocyanin-rich berries are food ingredients used in soft drinks and jellies and as natural food colorings [3]. Ripe elderberries (*Sambucus nigra* L.) are especially rich in anthocyanins and have been consumed as food and medicine since prehistoric times [4,5]. Currently elderberries are used as foods like jellies, jams and yogurts and as food complements such as juices, syrups and tablets; in addition, they are a source of vitamins and are active against flu and colds [6,7,8]. Furthermore, they have been used as a wine rich in health-promoting compounds [9]. Among the compounds found in elderberries are phenolic acids, flavonoids, vitamins, lectins and aroma compounds, etc. [6,8,10,11]. Low molecular weight compounds with pharmacological activity, for instance, the activation of the human peroxisome proliferator-activated receptor (PPAR) γ [12] and anti-diabetic effects [13] have been described. Therefore, taking into account the alleged properties of elderberries, they could be regarded as a functional food. Concentrated elderberry juices rich in flavonoids are commonly prepared for human consumption by expression of the berries with no additional thermal treatment, in order to preserve the stability of the antioxidant compounds [6,14]. Consequently, it is necessary to prepare juices that retain such healthy compounds and their properties.

Berries and other vegetables may also contain anti-nutritional factors like lectins (sugar-binding proteins) that could in some way affect their nutritional efficiency. However, despite the possibility that ingesting high concentrations of lectins could prove deleterious, many of these proteins, including RIPs (ribosome-inactivating proteins), have health-promoting effects that may be influential in the acknowledged benefits of eating certain fruits and vegetables [15,16]. The consumption of diets with small amounts of kidney beans, containing a high concentration of *Phaseolus vulgaris* lectins (PHA), promoted reversible gut growth and altered body composition, increasing the weight of the small intestine, pancreas, caecum and colon in rats [17]. Furthermore, the ingestion of *Phaseolus vulgaris* lectin induced reversible growth and maturation of the gastrointestinal tract in suckling rats [18].

The pioneering and seminal work of Van Damme and Peumans [19,20] revealed that together with the low molecular weight compounds, *Sambucaceae* species contain a number of lectins that share a high amino acid sequence homology. Some of these lectins have the *N*-glycosidase activity characteristic of type II ribosome-inactivating proteins or RIPs [21]. Common elderberry (*Sambucus nigra* L.) contains type II RIPs in bark (nigrin b-SNA V, SNA I and related proteins), fruits (nigrin f) and seeds (nigrin s) [5]. Dwarf elder (*Sambucus ebulus* L.) also contains type II RIPs referred to as ebulins in fruits (ebulin f), leaves (ebulin l), and rhizomes (ebulins r1 and r2) [22]. In addition to RIPs, *Sambucus* spp. contains D-galactose-binding lectins devoid of translational inhibitory activity [5,23]. Fruits likewise contain the monomeric lectin SNA IV [24], whose amino acid sequence shares a high degree of identity with SNA III from seeds and SNA II from bark [25,26].

Elderberry pollen, blossoms and fruits have been found to contain the allergen Sam n1 that triggers type I allergy [27]. Certain Sam n1 tryptic peptides display a high amino acid sequence with *Sambucus* lectins and type 2 RIPs [28,29]. This led to the hypothesis that the Sam n1 allergen could in fact be a member of a broad allergen family present in *Sambucus* [28]. *S. ebulus* lectins ebulin f and SELfd have been shown to be resistant to a simulated gastric fluid but highly sensitive after short-term heating [28]. This tallies with the general belief that resistance to gastric digestion correlates with the allergenicity of food proteins [30]. Nonetheless, gastric labile allergens and gastric resistant non-allergens have been reported [31,32]. Since the presence of gastric stable lectins in elderberry juices could determine hitherto unknown bioactive effects and allergies, we carried out studies aimed at reducing the presence of lectins without producing major effects on anthocyanin content and antioxidant properties. The objectives of the present research were to assess in vitro whether a short heat pretreatment of elderberry lectins and extracts could have a relevant influence on the digestibility of lectins by hydrolytic enzymes and on total phenol content, antioxidant and free-radical scavenging activities.

## 2. Results

### 2.1. Sensitivity of Elderberry Purified Lectins to Simulated Gastric Fluid

Elderberry juices are commonly prepared in such as a way as to avoid alteration, which could reduce their quality and organoleptic and color properties [6,14], and to decrease the levels of cyanogenic glycosides, which could be harmful. Since the fruits contain lectins, it was appropriate to assess digestibility in a simulated gastric fluid (SGF) containing pepsin, which has been described as a general in vitro procedure to assess digestibility and the connection with potential allergenicity [30].

**Figure 1:**
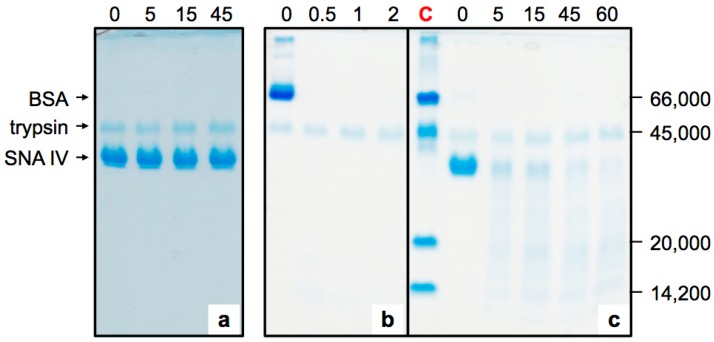
SDS-PAGE analysis of the sensitivity of *S. nigra* fruit lectin SNA IV. (**a**) Native SNA IV incubated with SGF at 37 °C for 0, 5, 15 or 45 min; (**b**) native BSA incubated with an SGF at 37 °C for 0, 0.5, 1 or 2 min; (**c**) heat-treated SNA IV incubated with simulated duodenal fluid (SDF) at 37 °C for 0, 5, 15, 45 or 60 min; After digestions the samples were analyzed by SDS-PAGE. C line, relative molecular mass (Mr) standards: 66,000 Da BSA; 45,000 Da ovalbumin; 20,000 Da trypsin inhibitor; 14,200 Da alpha-lactalbumin.

**Figure 2:**
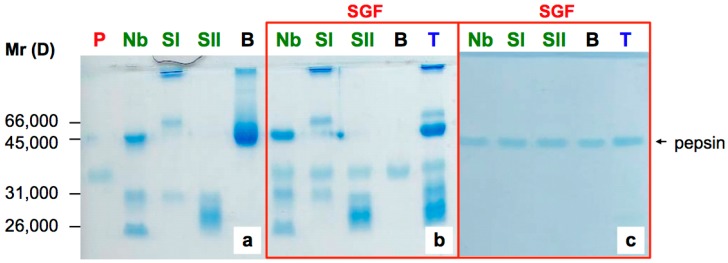
Sensitivity to pepsin of *S. nigra* lectins by SDS-PAGE analysis. (**a**) Native untreated lectins incubated at 37 °C; (**b**) native untreated lectins incubated at 37 °C with an SGF; (**c**) heat-treated lectins (10 min, 100 °C) incubated at 37 °C with an SGF. After digestions the samples were analyzed by SDS-PAGE. **P**: pepsin, **Nb**: nigrin b-SNA V, **SI**: SNA-I, **SII**: SNA-II, **B**: BSA, **T**: nigrin b-SNA V + SNA-I + SNA-II + BSA. Mr standards: 66,000 D BSA; 45,000 D ovalbumin; 31,000 D nigrin b-B chain; 26,000 D nigrin b-A chain.

For these experiments, pure native proteins were used (Figure 1 and Figure 2). Ripe elderberries essentially contain the monomeric SNA IV and a very small amount of the ribotoxin nigrin f [33]; nigrin s was absent since the seeds were removed intact during the preparation of the extracts [34]. As shown in Figure 1, SNA IV was completely stable upon incubation with an SGF at 37 °C for 45 min (Figure 1a); under these conditions pepsin did not undergo auto-digestion. BSA digestibility was also analyzed as an internal experimental control. After 30 s, all the protein was degraded (Figure 1b). In contrast, as shown in Figure 1c, upon heating in a boiling water bath for 5 min the lectin became sensitive to pepsin, and after 5 min of incubation a large amount of the denatured protein was degraded.

### 2.2. Sensitivity of Elderberry Lectins to Simulated Duodenal Fluid

**Figure 3:**
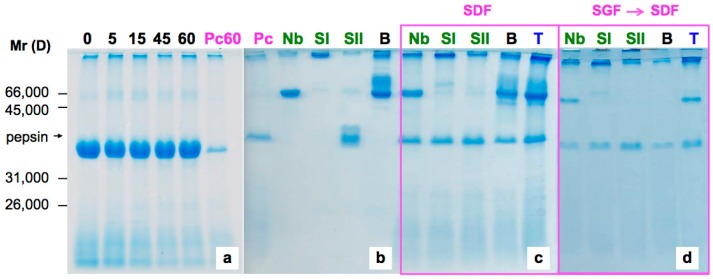
Sensitivity to SDF containing pancreatin of *S. nigra* lectins by SDS-PAGE analysis. (**a**) Native SNA IV incubated with SDF at 37 °C for 0, 5, 15, 45 or 60 min; (**b**) native untreated lectins incubated at 37 °C; (**c**) native untreated lectins incubated at 37 °C with SDF; (**d**) sequential digestion of native untreated lectins incubated at 37 °C, first digestion with SGF and after with SDF. Following digestions, the samples were analyzed by SDS-PAGE. **Pc60**: pancreatin incubated with SDF for 60 min, **Pc**: pancreatin, **Nb**: nigrin b-SNA V, **SI**: SNA-I, **SII**: SNA-II, **B**: BSA, **T**: nigrin b-SNA V + SNA-I + SNA-II + BSA. Mr standards: 66,000 D BSA; 45,000 D ovalbumin; 31,000 D nigrin b-B chain; 26,000 D nigrin b-A chain.

We next investigated the sensitivity of native elderberry lectin SNA IV to SDF. Pancreatin is the enzymatic component of the fluid. The preparation from porcine pancreas used in this study is a complex enzymatic mixture that contains amylase, trypsin, lipase, ribonuclease and protease. As shown in Figure 3, SNA IV was not sensitive to pancreatin after 60 min of incubation.

Practically the same lack of sensitivity was found with nigrin b, SNA I and SNA II (Figure 3b,c). These proteins formed large aggregates with low penetration into the 15% polyacrylamide gel; this was probably as a consequence of the combined action of proteases and amylase that could promote the appearance of new interactions between the polypeptides chains.

Our results also indicate that degradation of BSA was triggered only by the SGF. A two-step digestion, firstly SGF and then SDF, of the samples of native *S. nigra* lectins produced nearly the same results as digestion with SDF alone. As shown in Figure 3d, the sequential digestion did not affect nigrin b and promote the precipitation of undegraded material from SNA I and SNA II in the corresponding well. This indicates that, unless they are heat-pretreated, the native lectins are refractory to proteolysis and may, therefore, reach the small intestine mostly intact.

### 2.3. Effects of Heat Treatment on Total Phenol Content, Antioxidant and Free-Radical Scavenging Activities

Elderberry has been described as one of the berries richest in phenolic compounds such as phenolic acids and polyphenols [35]. Such compounds have been described as being sensitive to heat [36]. Since the preparation of berry juices is aimed at preserving as many antioxidant and health beneficial compounds as possible, we explored the effects of heat treatment of elderberry extracts on total phenol content, antioxidant and free-radical scavenging activities.

As shown in Table 1, ripe fruits contained nearly twice the amount of phenolics expressed as gallic acid equivalents (GAE) per gram of berry used to prepare the extract. Heat treatment had a considerable effect on content in both type of berries, after 40 min of incubation in a boiling water bath with between 50% and 65% reduction. In the case of free-radical scavenging activity, 40 min of incubation in boiling water was responsible for a similar decay in activity as with phenolics. Under these conditions the losses represented 60% and 53% in ripe and green berries, respectively. In the case of antioxidant activity, the effect was similar.

### 2.4. Effect of Heat on Anthocyanidins

Anthocyanins are water-soluble flavonoids characteristic of ripe elderberries [37]. Important elderberry effects such as antitumor influence are thought to be due to these anthocyanins [38]. Therefore, we explored the effects of heat treatment of extracts of ripe fruits on anthocyanins by the differential pH procedure. As shown in Table 1, total monomeric anthocyanin content expressed as cyanidine-3-glycoside was negligible in green fruits and underwent severe decay (72%) after 40 min of incubation in a boiling water bath. By contrast in ripe fruits, short incubation times like 10 min, reduced content by a mere 3%.

**Figure 4:**
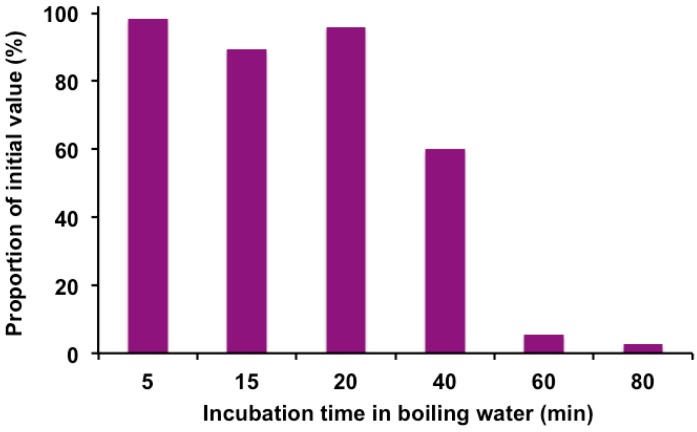
Effect of incubation in a boiling bath of ripe elderberry extracts on cyanidin-3-glucoside plus cyanidin-3-sambubioside content. The extracts were incubated for the times indicated and analyzed by HPLC, as indicated in the Materials and Methods section; peaks containing these anthocyanins were considered.

Cyanidin-3-glucoside (C-3-G) and cyanidin-3-sambubioside (C-3-Sam) are the most significant phenolics present in ripe elderberry [14,39,40]. We further investigated the specific effects of heat on C-3-G and C-3-Sam by HPLC. As shown in Figure 4, after 20 min of incubation in boiling water there was a 20% reduction and more than 90% after 40 min. Since both compounds move together in HLPC [14], the results are given for the mixture.

Heat treatment of the extracts for 20 min reduced by 20% the content of the mixture of C-3-G and C-3-sam anthocyanins, as with total monomeric anthocyanin content. After 40 min of heat treatment, losses represented 40%, and after 60 min of treatment only residual amounts were detected. It is worth noting that although 5 min of heat treatment produced almost no effect on anthocyanin content, it greatly increased the sensitivity of SNA IV, which is the most predominant lectin in elderberry fruits, to pepsin degradation (Figure 1).

## 3. Discussion

In elderberry juice preparation it is desirable to retain as many nutraceuticals, antioxidant and free-radical scavenging activities of the raw berry material as possible, with the lowest content of toxic-like cyanogenic glycosides, anti-nutritional factors and/or protein toxins such as lectins. Ripe elderberry fruits contain large amounts of the monomeric lectin SNA IV together with a vestigial presence of the type 2 RIP nigrin f-SNA Vf [34]. Furthermore, they contain several proteins that react with sera from patients suffering type I allergy [27]. One such allergen, Sam n1, was isolated and partially characterized as a polypeptide chain with Mr 33,200 D; it contained amino acid sequences with a high degree of similarity to sequences of lectins and RIPs isolated from *S. nigra* and *S. ebulus* lectins [27,28,29]. This posits the question as to whether Sam n1 is a lectin or a type II RIP itself, a question that remains to be answered. Furthermore, it raises the hypothesis that elderberry lectins are also in fact allergens belonging to a broad family of which Sam n1 is one of them. Another question related with the ingestion of elderberry native lectins is the potential effect on the growth of mucosa cells. It has been described that ingestion by rats of SNA II like the PHA lectin may act as growth factors which promoted small intestinal growth [18,41]. Lectins may trigger both deleterious and health–promoting effects. It is rather difficult to distinguish between them since we have not a picture on the lectin action alone. Nonetheless, it is clear that raw elderberries ingestion leads to the ingestion of allergens and lectins, and that the final result will be a balance between such effects. While the elimination of allergens may be desirable the elimination of lectins with positive effects on health must be avoidable. Therefore, the ingestion of native elderberry lectins could raise concerns in terms of its role as an allergen and as a growth factor for the small intestine. At present, we have not an answer to that open question.

Even though our study is limited to the in vitro conditions, the finding that short heat treatment of elderberry lectins sensitize it to pepsin attack in a simulated gastric fluid without important losses of total monomeric anthocyanins, is important as far as fruit preparations are concerned. It is desirable that the extracts retain the healthy properties resulting from anthocyanins without any deleterious effects due to the presence of intact potentially toxic or allergenic proteins. It has been reported that native nigrin b-SNA V is resistant to acidic pepsin [42]. The present research confirms this finding and extends the analysis to other *Sambucus* lectins, in particular those present in fruits. We have found in this regard that elderberry bark lectins are somewhat more sensitive to moist heat sensitizing treatment to pepsin than SNA IV present in ripe fruits. Furthermore, elderberry lectins were poorly degraded by pancreatin, which suggests that if they pass intact through the gastric compartment to the duodenum they will be undigested.

The transfer of intact lectins to the small intestine would have the capacity to challenge the gastrointestinal immune system and thus trigger an undesired response in already sensitized subjects bearing type I allergy to Sam n1. Consequently, the barrier for the potentially deleterious effects of lectins must be the stomach, where acidic pepsin acts. From an allergenic point of view, it is important to set up a procedure to permit elimination of lectins in the gastric digestion without affecting the content of nutraceuticals such as phenolics and other antioxidant and free-radical scavenging compounds. Treatment of purified elderberry lectins in a boiling water bath for 5–10 min resulted in all lectins becoming sensitive to acid pepsin. A similar effect of moist heat able to sensitize to pepsin was seen on other proteins like phaseolin [43], and anacardein [44].

Under our heat treatment conditions, neither total phenol content nor free radical scavenging activities and antioxidant activities were largely affected (reductions of 10% or lower after 10 min of incubation in a boiling water bath). The assays of phenolics with the Folin-Ciocalteau’s phenol reagent led to overestimation compared with detection by HPLC [14]. Nonetheless, as we wanted to state the effects of heat treatment on reactivity compared with the control untreated extracts, the results give a good picture of heat sensitivity of the mixture of compounds that reacted under such conditions. The analysis of total anthocyanins indicated that there were no significant changes in the content of the heat–treated extracts after short incubations. This is especially important, since C-3-G has been reported as a chemopreventive and chemotherapeutic agent in cancer tumor development [38]. Furthermore, as safety concerns, our results are strengthened by the recent finding that processing elderberry for liquor preparation reduced cyanogenic glycosides by up to 96% [39].

In light of the present results, short heat treatment of *S. nigra* raw extracts, under our conditions, is important for: (a) elimination of potential food allergy risks due to allergenic *Sambucus* proteins; (b) elimination of potential abnormal growth effects of lectins due to ingestion of raw elderberry juices; (c) preservation of the antioxidant capacity; (d) reduction of cyanogenic glycosides; (e) partial sterilization of the juices. These advantages should be taken into account from the plant food safety and industrial points of view in the production of juices [6,9,39]. To date more research is needed to establish whether elderberry ingestion would have potential health-promoting effects mediated by lectins. In vivo studies in rodents will be performed to address lectin digestibility and how it could be affected by food matrices. In addition, the oxidative status of plasma and the different tissues will be also studied after ingestion of either raw or processed elderberry extracts. However, since elderberry allergies based on the allergen Sam n1 with a high amino acid sequence homology with elderberry lectins have been reported, it seems prudent to ensure ingestion of preparation with a low content of these proteins, at least until deeper studies, including in vivo experiments, posed the lack of long-time toxicity of elderberry based beverages.

## 4. Materials and Methods

### 4.1. Materials

All chemicals and biochemicals were of the highest purity available. Pepsin from porcine gastric mucosa (ref. P7000) and pancreatin from porcine pancreas (ref. P7545) were obtained from Sigma (St. Louis, MO, USA). A ready-to-use acrylamide (37.5%)-bisacrylamide (0.8%) mixture was obtained from Amresco (Solon Ind., Solon, OH, USA). Bark and ripe fruits of elderberry (*Sambucus nigra* L.) were collected from well characterized wild trees (Mansilla de las Mulas, León, Spain) in mid-August and early September, respectively. All samples were stored and frozen at −20 °C. SNA I, nigrin b-SNA V, and SNA II were isolated by affinity chromatography on acid-treated Sepharose 6B (GE Healthcare, Barcelona, Spain), as described elsewhere [45]. A preparation of SNA IV containing small amounts of nigrin f was prepared from ripe fruits by affinity chromatography, as mentioned previously [33]. All purified proteins were tested for human red blood cell agglutination, in line with existing reports [28]. The blood samples were obtained from the Hematology Department of the Hospital Universitario del Río Hortega by Dr. Rosario del Villar. They were washed with a solution of 0.14 M NaCl and 0.005 M Na-phosphate (pH = 7.5); following hematocrit determination it was concentrated to 1% (*v*/*v*) by mild centrifugation.

### 4.2. Preparation and Heat Treatment of Elderberry Extracts

Extracts from *S. nigra* L. were prepared by grinding frozen samples of fruits with a buffer solution containing 0.28 M NaCl and 5 mM sodium phosphate (pH = 7.4), for 4–5 min at room temperature. The paste was filtered through a double layer of nylon tissue, and then centrifuged twice at 7500× *g* for 30 min at 4 °C. The resulting extract was stored at −20 °C until use. Elderberry extracts were incubated in a boiling water bath in cap-closed tubes and were subsequently placed in an ice-water bath and used immediately.

### 4.3. Pepsin Digestion

The simulated gastric fluid reaction mixtures (90 µL) contained the following: 0.3 mg of pepsin, 0.066 N HCl, and 30 mM NaCl along with the test protein. Incubations were carried out at 37 °C for the times indicated in the legends of the figures. Digestion was halted by adding 30 µL of 200 mM NaHCO_3_ (pH = 11). From these mixtures 18 µL were extracted and mixed with 6 µL of four-times concentrated loading buffer containing 0.260 M Tris (pH = 6.8), 40% glycerol, 8.4% sodium dodecyl sulfate, and 0.042% bromophenol blue, and analyzed by SDS-PAGE. Experiments on digestion by SGF were carried out five times.

### 4.4. Pancreatin Digestion and Sequential Digestion

The simulated duodenal fluid reaction mixtures of 90 µL contained: 0.78 mg of pancreatin and 0.05 M KH_2_PO_4_ (pH = 6.5) apart from the test protein. Incubations were carried out at 37 °C for the times indicated in the legends of the figures. Digestion was halted at appropriate times by incubation in a boiling water bath, and from these mixtures 18 µL were set aside and mixed with 6 µL of four-times concentrated loading buffer, as for SDF digestion; once again, SDS-PAGE analysis was performed. The sequential digestion was carried out with samples from pepsin digestion neutralized with sodium carbonate as indicated above for pepsin digestion and supplemented with the same concentration of pancreatin as for pancreatin digestion, followed by incubation in the same conditions as for digestion of pancreatin only. Experiments on digestion were carried out three times.

### 4.5. SDS-Polyacrylamide Gel Electrophoresis

The protein study by SDS-PAGE [46] was conducted with the MiniVE minigel (10 cm × 8 cm × 7.5 mm) system (GE Healthcare, Barcelona, Spain), using 4% staking gel and 15% polyacrylamide separation slab gels. The samples were incubated for 5 min at 100 °C in the loading buffer (65 mM Tris-HCl (pH = 6.8), 2% (*w*/*v*) SDS, 10% (*v*/*v*) glycerol and 0.0105% (*w*/*v*) bromophenol blue). A volume of 18 µL containing the protein sample was loaded into each well and electrophoresis was carried out, at 20 °C and 25 mA per gel, by means of a buffer with 25 mM Tris-HCl (pH = 8.3), 192 mM glycine, and 0.1% (*w*/*v*) SDS. Gels were stained overnight and unstained, as reported previously [28].

### 4.6. Total Phenol Determination

Total phenol compounds present in elderberry extracts were determined with the Folin-Ciocalteau’s reagent for phenols as follows: 1.5 mL of sample was mixed with 0.6 mL of saturated sodium carbonate and 0.2 mL of Folin-Ciocalteau’s reagent. The tubes were incubated at 50 °C for 10 min, cooled and A_760_ was read. Gallic acid was used as the standard for calibration. The results were expressed as gallic acid equivalents per gram of elderberry used for extract. The number of experiments was three and the experimental points were conducted by triplicate.

### 4.7. CUPRAC Assay for Antioxidant Activity

Antioxidant activity was determined by the CUPRAC assay [47]. Reaction mixtures of 4 mL contained: 0.02 mL of sample, 1 mL of 10 mM CuCl_2_ and 1 mL of 7.5 mM neocuproin in ethanol, and 1 mL of 1 M ammonium acetate (pH = 7.0). The mixture was kept 60 min to allow color development, after which A_450_ was read. Gallic acid was used as the standard for calibration. The results were expressed as gallic acid equivalents per gram of elderberry used for extract. The number of experiments was three and the experimental points were conducted by triplicate.

### 4.8. DPPH Radical-Scavenging Activity

The capacity to scavenge free-radicals was assessed by 2,2-diphenyl-1-picrylhydrazyl (DPPH) color decay after 10 min of incubation [48]. Reaction mixtures of 3 mL contained: 0.03 mL of sample and 2.97 mL of 0.1 mM DPPH dissolved in methanol. These were kept for 10 min in the dark for color development, and the reduction in color was determined by measuring A_515_. Trolox was used as the standard and results were expressed as Trolox equivalents per gram of elderberry used for extract preparation. The number of experiments was three and the experimental points were conducted by triplicate.

### 4.9. Total Anthocyanin Analysis

Total anthocyanidins were determined by the differential pH procedure [49]. Two mixtures of 1.25 mL of different pH containing the samples were prepared. One contained 0.2 M KCl adjusted to pH 1 with HCl acid. The other contained 0.2 M sodium acetate adjusted to pH 4.5 with acetic acid. The A_510_ and A_700_ at pH 1 and 4.5 were determined and a resulting A* was calculated using the equation: A* = (A_510_ − A_700_)^pH 1.0^ − (A_510_ − A_700_)^pH 4.5^. Finally, the monomeric anthocyanidin (A_M_) was calculated with the equation: A_M_ (mg/L) = A*·Mr*·dilution factor·1000/26,900 L·mol^−1^·cm^−1^ (molar extinction coefficient). Cyanidine-3-glycoside was used as the standard. The number of experiments was three and the experimental points were conducted by triplicate.

### 4.10. HPLC Analysis of Anthocyanins

Elderberry extracts was assayed by HPLC/UV-Vis with a Spherisorb ODS-1 C18 (5 µm, 250.0 × 4.6 mm) column (Waters Cromatografía, S.A., Cerdanyola del Vallès, Spain) coupled with a 4 × 3 mm C18 guard column (Phenomenex, Torrance, CA, USA); acetonitrile and 1% (*v*/*v*) phosphoric acid-10% (*v*/*v*) acetic acid ultrapure water were used as eluent in accordance with the following gradient: from 2% to 20% of acetonitrile for 10 min, and from 20% to 2% of the same solvent for 15 min. The retention time for the main peaks was around 12.5 and 16.9 min, with a flow rate of 1.0 mL/min. Eluent absorbance was monitored at a wavelength of 520 nm [50]. The experimental analysis was carried out twice.

## 5. Conclusions

Lectins present in elderberries are resistant to in vitro degradation by simulated gastric and intestinal fluids. Brief incubation in a boiling water bath however, made them completely sensitive to pepsin digestion; this permitted complete hydrolysis in a simulated gastric fluid and the reduction of risks associated with potential allergenicity resulting from allergens such as Sam n1 and structurally-related lectins, in particular SNA IV, present in ripe fruits during elderberry juice processing. This brief heat treatment reduced by less than 10% the content of total anthocyanidins, total compounds reacting with the Folin-Ciocalteau’s phenols reagent and free-radical scavenging activity of the elderberry. The presented results thus suggest a way to improve juice safety without altering the valuable nutraceutical properties of this fruit.

## Figures and Tables

**Table 1:** Influence of boiling time of fruits extracts on: (**A**) total phenol content [gallic acid equivalents (µg)/wet weight of fruit (g)]; (**B**) free radical scavenging activities [Trolox equivalents (µg)/wet weight of fruit (g)]; (**C**) antioxidant activities [gallic acid equivalents (µg)/wet weight of fruit (g)] and (**D**) total monomeric anthocyanins content [Cyanidine-3-glycoside equivalents (µg)/wet weight of fruit (g)]; data are expressed as mean ± confidence interval (*p* < 0.05, *n* = 3).

EXTRACT	Time (min)	A	B	C	D
Green fruits	0	4482.8 ± 94.4	0.79 ± 0.01	370.7 ± 3.1	83.5 ± 5.5
	10	4314.0 ± 99.6	0.71 ± 0.02	360.8 ± 2.1	76.0 ± 6.8
	20	3993.0 ± 67.9	0.59 ± 0.01	327.0 ± 2.5	62.1 ± 5.6
	40	2110.2 ± 67.1	0.37 ± 0.02	174.4 ± 7.9	23.0 ± 4.6
	80	571.0 ± 43.5	0.19 ± 0.01	118.8 ± 18.0	7.5 ± 4.8
Ripe fruits	0	8928.0 ± 84.4	9.10 ± 0.33	4821.4 ± 42.4	2704.5 ± 5.5
	10	8795.0 ± 138.7	8.61 ± 0.23	4776.8 ± 57.5	2625.5 ± 6.8
	20	8617.0 ± 296.9	7.87 ± 0.30	4295.4 ± 247.7	2185.4 ± 5.6
	40	5491.9 ± 54.1	3.55 ± 0.10	2055.9 ± 224.1	746.4 ± 4.6
	80	2885.9 ± 111.6	2.92 ± 0.08	1380.1 ± 45.4	404.6 ± 4.8
